# Assessing Biomedical and Psychosocial Factors in a Cross-Sectional Study of School Dropouts Among Pregnant Adolescents and Young Mothers in Quito, Ecuador

**DOI:** 10.7759/cureus.55958

**Published:** 2024-03-11

**Authors:** Jorge A Naranjo, Doris E Arevalo, Juan D Naranjo-Vinueza, Brenda A Vacas, Mireya Salcedo, Sofía M Borja, Rubén L Gallegos

**Affiliations:** 1 Department of Teaching, Faculty of Psychology, Central University of Ecuador, Quito, ECU; 2 Department of Psychology/Comprehensive Care Service for Adolescents, Hospital Gineco-Obstétrico Isidro Ayora, Quito, ECU; 3 Department of Teaching/Psychiatry, Instituto Nacional de Psiquiatría, Mexico City, MEX; 4 Department of Social Work/Comprehensive Care Service for Adolescents, Hospital Gineco-Obstétrico Isidro Ayora, Quito, ECU

**Keywords:** school dropout, women's and adolescent health, public health education, education, risk factors, adolescent motherhood, adolescent pregnancy

## Abstract

Background: Over nearly three decades, Ecuador experienced a significant rise in adolescent motherhood.

Objectives: By focusing on social, health, and psychological aspects, the research aims to reveal the complex factors influencing the decision to discontinue education. The emphasis on providing a platform for direct expression of personal experiences not only adds qualitative depth to the study but also ensures that the voices of those involved are heard authentically.

Methods: Employing a nonexperimental, descriptive, cross-sectional approach with qualitative and quantitative methods, the research delves into the interplay of biological, psychological, and social factors. Descriptive statistics, presented through tables and graphs, were used for variable analysis, complemented by inferential statistics to validate hypotheses. Focus group sessions, processed with ATLAS.ti (ATLAS.ti Scientific Software Development GmbH, Berlin, Germany) underwent a thorough review in workshops with Servicio de Atención Integral para Adolescentes (SAIA) experts. Adolescent participants were randomly recruited through the hospital's system.

Results: The findings unveiled a significant dropout rate among adolescents, where pregnancy was just one factor influencing their decision. Those discontinuing education often embraced a life project centered on motherhood and domestic roles, facing domestic violence and mental health disorders. In contrast, those persisting with education were driven by professional development, facing challenges but benefiting from family support. Despite unwanted pregnancies and low contraceptive use, many found personal growth and identity affirmation in motherhood.

Conclusions: Our research highlights key insights into factors like pregnancy desire, contraception, reactions, and challenges. Urgent action is needed to address systemic problems and provide holistic support, acknowledging the resilience and validity of choices made by adolescent mothers in balancing motherhood with education and career goals.

## Introduction

Background and rationale

Adolescence, the stage of life between the ages of 10 and 19 [1], represents a significant developmental period characterized by profound physical changes, particularly cerebral and sexual development. This complex psychosocial evolution enhances capacities for abstract reasoning, facilitates the pursuit and assertion of identity, supports the transition to social and economic independence, and enables the acquisition of roles and skills necessary for adult responsibilities [2]. One crucial realm of exploration during this stage pertains to sexuality, which is frequently shrouded in myths, taboos, and stereotypes that elevate risk and vulnerability for adolescents [3]. The lack of sex education and insufficient access to health services often results in adolescent pregnancies [4]. In 1990, Ecuador reported 40,218 live births from adolescent mothers, with 634 births from women aged 10 to 14 years and 39,584 from those aged 15 to 19 years. This number increased by 2018, when there were 56,267 live births, with 2,099 from the 10 to 14 age group and 54,168 from those aged 15 to 19 years old [5].

School dropout has numerous causes that are both social and individual. Poverty often leads to early entry into the workforce. Violent or neglectful parenting styles devalue education, as parental involvement is directly proportional to academic performance [6]. Inadequate support from teachers, institutions, and educational policies, can increase dropout rates [7]. Students with early investments in skills, from parents or programs benefit more from staying in school [8]. Subpar education systems that effectively alienate students are among these factors. Bullying and teasing environments affect students, influencing their choice to leave school [9]. As well, poor mental health is closely linked to dropout rates. Notably, high school dropouts are significantly more likely to report recent suicide attempts compared to high school graduates [10]. A 2010 survey conducted by the National Institute of Statistics and Censuses of Ecuador, the entity responsible for collecting, processing, and disseminating official statistical information in Ecuador indicated that only 22.1% of adolescent mothers attend school regularly, while 77.9% had dropped out, resulting in 1.8 to 2.8 fewer years of education among these adolescent mothers [5]. There is a close relationship between teenage pregnancy and school desertion. Adolescents who deserted from school before pregnancy are more vulnerable [11], since they are affected by obstetric or biomedical problems, psychological difficulties (depression, anxiety, confused life project), and socio-family conditions (shame, bullying, discrimination, economic dependence [12], migration, low family educational level).

According to a study carried out by the Ministry of Public Health of Ecuador, 6,487 adolescents dropped out of school due to pregnancy, being the fourth cause of school dropout. More than half (56%) were in basic education at the time, giving a total of 36,871 years of schooling lost, with an average of 5.8 years per woman, equivalent to $728.5 million of lost income that they would have received had they not dropped out of school before the higher level [13].

Many pregnant adolescents are compelled to leave school, disrupting their life plans and reducing their opportunities to enter the labor market, leading to lower productivity and income [14]. Studies have shown that income levels among women who became mothers during adolescence are substantially lower than those of women who delayed motherhood until adulthood, primarily due to educational disparities [15].

Ensuring the retention of girls and adolescents in the education system significantly reduces pregnancy rates, enhances decision-making capabilities, and improves women’s life prospects [16]. Identifying the risk factors leading to school dropout due to pregnancy and motherhood is crucial, mitigating the impact of these circumstances (tertiary prevention) and facilitating school reintegration or redesign of life projects (quaternary prevention). This exploratory study represents a valuable initiative in addressing the multifaceted challenges faced by pregnant adolescents and adolescent mothers who contemplating leaving school. By focusing on social, health, and psychological aspects, the research aims to reveal the complex factors influencing the decision to discontinue education. The emphasis on providing a platform for direct expression of personal experiences not only adds qualitative depth to the study but also ensures that the voices of those involved are heard authentically.

## Materials and methods

A nonexperimental, descriptive, cross-sectional study incorporating both qualitative and quantitative approaches was implemented. By combining qualitative and quantitative methods, the research aims to gain a comprehensive understanding of the factors influencing school dropout. We propose the hypothesis that the interaction of biological, psychological, and social factors significantly influences the occurrence of school dropout among pregnant adolescents and mothers. Our study explores a comprehensive set of indicators, as outlined in Table 1. Within biological risk factors, the impact of symptomatology related to physical discomfort, past medical history, and child health problems was investigated. Psychological risk factors are explored through an examination of personal factors such as pregnant desire, contraceptive use, and reactions to pregnancy, as well as family and peer attitudes towards pregnancy and motherhood. Social characteristics, including marital status, nationality, occupation, and educational conditions (such as type of school, study modality, and duration), are also considered.

**Table 1:** An operational array of variables, categories, and indicators

Units of Analysis	Category	Indicator
Pregnancy in adolescence	Pregnancy characteristic	Age
		Gestation time
		Pregnancy desire
		Use of contraceptives
Maternity	Maternity characteristics	Age
		Number of children
		Current child's age
School dropout and life project	Educational characteristics	Approved years
		School attendance
		Current school
		Study mode
		Study schedule
		Study desire
		Vocational decision
		Time since dropout
	Biological reasons for school dropout	Physical discomfort
		Past medical history
		Child's health problems
	Psychological and social reason for school dropout	Personal psychological problems
		Family altitude
		Partner attitude
		Peers attitude
		School institution attitude
		Knowledge of rights
		Family dependence level
	Family conditions	Family dependence level
		Marital status
		Relationship with partner
		Income level
		Current occupation

The strengths lie in qualitative data providing depth and context, capturing both numerical trends and qualitative insights, facilitating efficient data collection, and enabling hypothesis generation. The study acknowledges limitations, including challenges in establishing cause-and-effect relationships, potentially limited generalizability, and the subjective nature of qualitative data. Despite these limitations, the research aspires to offer valuable insights reflective of real-world conditions, shedding light on the dynamics of school dropout among pregnant adolescents and young mothers in Quito, Ecuador.

Our multidisciplinary team, which comprised doctors, psychologists, social workers, and students from "Servicio de Atención Integral para Adolescentes” (SAIA) at the Hospital Gineco-Obstétrico “Isidro Ayora” (HGOIA), and the Educational Psychology Career of the Universidad Central del Ecuador designed and validated by expert opinion a survey administered to each participant. Participants were pregnant adolescents and adolescent mothers with children under the age of one year who attended SAIA in Quito, Ecuador, between July and October 2019.

Before participating, individuals received detailed information about the research, including its title, objectives, and purposes. They were informed about potential risks and benefits, with an explanation of the project methodology in clear, non-technical language. Participants read the informed consent letter, were encouraged to ask questions, and were reminded of the voluntary nature of their involvement, with the right to withdraw at any time. Both participants and their legal guardians provided their informed consent. The Institutional Review Board "Comité de Etica e Investigación del Hospital Gíneco-Obstétrico Isidro Ayora" approved our study protocol (Approval No. CIF5-CS-FF-2).

For confidentiality, each participant was assigned an alphanumeric code, and no personal identification data were collected. The principal researcher held sole access to and responsibility for safeguarding participant information throughout the study. Our team was responsible for data collection. Six team members specifically trained for the task inputted the data into an Excel database accessible to our researchers.

The survey was conducted in a private office and completed through individual interviews conducted by trained researchers. Over four months, from July to October 2019, we conducted an average of 10 interviews daily, divided equally between the inpatient and outpatient units, each lasting about 15 minutes. After data collection, these researchers received immediate supervision from the SAIA coordinator to clarify potential errors during the filling process. The final database was carefully checked and cleaned to eliminate data inconsistencies or participants whose responses indicated inconsistencies in survey completion.

Participants

We enlisted participants from both the outpatient and inpatient units at SAIA at HGOIA based on their presence in the clinic during our study. Our inclusion criteria targeted pregnant adolescents and adolescent mothers aged 12 to 19 who had discontinued their education during or after pregnancy. We excluded those who declined participation and those who had not been enrolled in school before their pregnancy.

A total of 391 participants were included in the final analysis. The participants comprised 100 (25.6%) pregnant adolescents and 291 (74.4%) adolescent mothers at the time of the investigation. The participants ranged from 12 to 19 years, with an average age of 16.8 years. Thirty-two participants (8.2%) were younger than 14, while 359 (91.8%) were between 15 and 19 years old.

We also conducted two focus group sessions involving 17 participants for a more qualitative approach. The first group comprised nine participants who were pregnant adolescents and adolescent mothers who had dropped out of school, while the second group included eight participants who were pregnant adolescents and adolescent mothers who remained in school. Each session took place in a separate room at SAIA and lasted three hours. We obtained prior consent from participants to record both video and audio during these sessions.

Statistical analysis

We used descriptive statistics to analyze the variables, including measures of central tendency such as frequency distribution, means, and percentages. The results are presented in tables, graphs, and intervals. Inferential statistics were used to verify the hypotheses.

We calculated our sample population using a 2017 database from SAIA, which showed 14,208 consults, of which 6,166 were pediatric (adolescent mothers), and 8,082 were OB-GYN (pregnant adolescents). On average, 26 and 32 consults were attended daily, respectively. Our sample calculation for a finite population used a confidence level of 0.95, a margin of error of 0.05, and an expected value of 0.50, indicating a required sample size of 375 individuals. We aimed to recruit 10% more individuals to account for potential statistical losses, resulting in a final target of 412 participants.

Following data collection, we transcribed the focus group sessions and processed the information in ATLAS.ti (ATLAS.ti Scientific Software Development GmbH, Berlin, Germany) based on variable categories and indicators. Our team and experts from SAIA conducted a rigorous review of the survey design and validation through two workshops; each question was subjected to thorough analysis. We randomly recruited adolescent participants through the hospital’s appointment and admission system.

## Results

Descriptive data

Of the 391 participants, 187 (47.8%) were still enrolled in school at the time of the interview, and 204 (52.2%) had ceased their education. The overall mean age of participants was 16.8 years, with a mean age of 16.4 years for students and 17.2 years for non-students.

Main results

Marital Status and Education

Most of the participants identified as single (58.6%), followed by those in civil unions (40.2%), and married (1.3%), as illustrated in Table 2. None of the participants reported being divorced, separated, or widowed.

**Table 2:** Marital status, nationality, and education of adolescents

Variable	Status	Study Currently					
		Yes		No		Total	
		N	%	n	%	n	%
Marital status	Single	138	35.3	91	23.3	229	58.6
	Married	2	0.5	3	0.8	5	1.3
	Civil Union	47	12.0	110	28.1	157	40.2
	Total	187	47.8	204	52.2	391	100.0
Nationality	Ecuadorian	181	46.3	180	46.0	361	92.3
	Other	6	1.5	24	6.1	30	7.7
	Total	187	47.8	204	52.2	391	100.0
Occupation	Private employee	0	0.0	8	2.0	8	2.0
	Public Employee	3	0.8	3	0.8	6	1.5
	Student	174	44.5	0	0.0	174	44.5
	Own business	1	0.3	3	0.8	4	1.0
	Family business	1	0.3	5	1.3	6	1.5
	Housewife/Homemaker	8	2.0	99	25.3	107	27.4
	Unemployed	0	0.0	18	4.6	18	4.6
	Other	0	0.0	5	1.3	5	1.3
	None	0	0.0	63	16.1	63	16.1
	Total	187	47.8	204	52.2	391	100.0
Variable	Status	Study Currently					
		Yes		No		Total	
		N	%	n	%	n	%
Marital status	Single	138	35.3	91	23.3	229	58.6
	Married	2	0.5	3	0.8	5	1.3
	Civil Union	47	12.0	110	28.1	157	40.2
	Total	187	47.8	204	52.2	391	100.0
Nationality	Ecuadorian	181	46.3	180	46.0	361	92.3
	Other	6	1.5	24	6.1	30	7.7
	Total	187	47.8	204	52.2	391	100.0
Occupation	Private employee	0	0.0	8	2.0	8	2.0
	Public Employee	3	0.8	3	0.8	6	1.5
	Student	174	44.5	0	0.0	174	44.5
	Own business	1	0.3	3	0.8	4	1.0
	Family business	1	0.3	5	1.3	6	1.5
	Housewife/Homemaker	8	2.0	99	25.3	107	27.4
	Unemployed	0	0.0	18	4.6	18	4.6
	Other	0	0.0	5	1.3	5	1.3
	None	0	0.0	63	16.1	63	16.1
	Total	187	47.8	204	52.2	391	100.0

A total of 35.3% of the studying pregnant adolescents reported being single, compared to 23.3% of non-students. Those in civil unions comprised 28.1% and 12.0% of the student and non-student groups in the total sample, respectively. Only 0.5% of studying pregnant adolescents were married, compared to 0.8% of non-students.

Nationality and Education

Most participants were Ecuadorian (n = 361; 92.3%), while 30 (7.7%) participants were from other countries (23 Venezuelan, four Colombian, and three Spanish). Among non-student pregnant adolescents, 46.0% were Ecuadorian compared to 6.1% foreigners.

Occupation and Education

Among those still studying, most reported being students as their primary occupation (n = 174; 44.5%), with the remainder engaged in other activities like housework (4.3%), public employment (0.8%), or self/family-owned businesses (0.3%). The primary occupation for those not in school was housework (25.3%), not looking for a job (16.1%), unemployed (4.6%), private employee (2.0%), family business (1.3%), public employment or owned businesses (0.8%) or other occupation (1.3%).

Education Characteristics

Table 3 shows that most student and non-student pregnant adolescents (n = 327; 83.6%) were enrolled in public educational institutions. Others attended private schools (n = 45; 11.5%), municipal schools (n = 16; 4.1%), or semi-public institutions (n = 3; 0.8%). Most pregnant students (n = 373; 95.4%) attended on-campus, distance studies (2.6%), and blended learning (1.8%). No girls have online studies.

**Table 3:** Educational characteristics

Variable	Type	Where Do You Study?		Where Did You Study?		Total	
		n	%	n	%	n	%
Type of school	Public	150	38.4	177	45.3	327	83.6
	Private	24	6.1	21	5.4	45	11.5
	Municipal	10	2.6	6	1.5	16	4.1
	Semi-public	3	0.8	0	0.0	3	0.8
	Total	187	47.8	204	52.2	391	100.0
School modality	On-campus	171	43.7	202	51.7	373	95.4
	Blended	6	1.5	1	0.3	7	1.8
	Distance	9	2.3	1	0.3	10	2.6
	Online	0	0.0	0	0.0	0	0.0
	Others	1	0.3	0	0.0	1	0.3
	Total	187	47.8	204	52.2	391	100.0
Time of study	Morning	98	25.1	121	30.9	219	56.0
	Afternoon	68	17.4	73	18.7	141	36.1
	Night	20	5.1	9	2.3	29	7.4
	No schedule	1	0.3	1	0.3	2	0.5
	Total	187	47.8	204	52.2	391	100.0

Off-campus studies were higher in young women who still study (blended 1.5%, distance 2.3%) compared to those who no longer study (blended 0.3%, distance 0.3%).

The time of study of the adolescents was higher in the morning (56.0%) than in the afternoon (36.1%) and evening (7.4%). Being the night schedule greater in students (5.1%) than in non-students (2.3%).

Pregnancy Desire, Contraception, and Pregnancy Reaction

Most participants (n = 303; 77.5%) had not planned to become pregnant and had not used any contraception (n = 308; 78.8%). Adolescents who were in school were significantly less likely (n = 26, 13.9%) to desire pregnancy compared to those who were not in school (n = 62, 30.4%; p < .001). The average time between pregnancy and school dropout was 17.8 months for those who desired pregnancy and 13.5 months for those who did not. Only 83 (21.2%) participants reported contraceptive use. A significant difference was found in contraceptive use between students (n = 41, 21.9%) and non-students (n = 42, 20.6%; p < .001).

Upon discovering their pregnancies, 48.3% (n = 189) of the participants reported fear, a response significantly more prevalent in students (n = 104, 55.6%) compared to non-students (n = 85, 41.7%; p < 0.002). Acceptance was the second most common reaction (33%, n = 129), being more common among non-students (N = 78; 38.2%) than students (n = 51; 27.3%). These differences were statistically significant (p 0.002). Additional reactions to pregnancy are outlined in Table 4.

**Table 4:** Pregnancy characteristics and studies

Variable	Type	Study Currently						P-Value
		Yes		No		Total		
		n	%	n	%	n	%	
Pregnancy desire	Yes	26	6.6	62	15.9	88	22.5	0.000
	No	161	41.2	142	36.3	303	77.5	
	Total	187	47.8	204	52.2	391	100.0	
Contraception	Yes	41	10.5	42	10.7	83	21.2	0.000
	No	146	37.3	162	41.4	308	78.8	
	Total	187	47.8	204	52.2	391	100.0	
Reaction to pregnancy	Acceptance	51	13.0	78	19.9	129	33.0	0.002
	Guilt	5	1.3	6	1.5	11	2.8	
	Fear	104	26.6	85	21.7	189	48.3	
	Illusion	9	2.3	25	6.4	34	8.7	
	Anger	1	0.3	4	1.0	5	1.3	
	Rejection	10	2.6	3	0.8	13	3.3	
	Indifference	1	0.3	0	0.0	1	0.3	
	Other	6	1.5	3	0.8	9	2.3	
	Total	187	47.8	204	52.2	391	100.0	
Type of reaction to pregnancy	Negative	120	30.7	98	25.1	218	55.8	0.001
	Neutral	7	1.8	3	0.8	10	2.6	
	Positive	60	15.3	103	26.3	163	41.7	
	Total	187	47.8	204	52.2	391	100.0	

In general terms, the type of reaction that the girl has towards pregnancy was examined. Adolescents who had a positive reaction to pregnancy (acceptance, excitement) constituted 163 individuals (26.3%), contrasting with those who had a negative attitude (n = 218; 55.8%) or a neutral and unclear response (n = 10; 2.6%).

Of the young women with a positive response, 15.3% were still studying, compared to 26.3% who had dropped out of school. Among adolescents with a negative attitude, 30.7% remained in school, while 25.1% had discontinued their education. These differences were found to be statistically significant (p = 0.001).

Principal Problems

**Figure 1:**
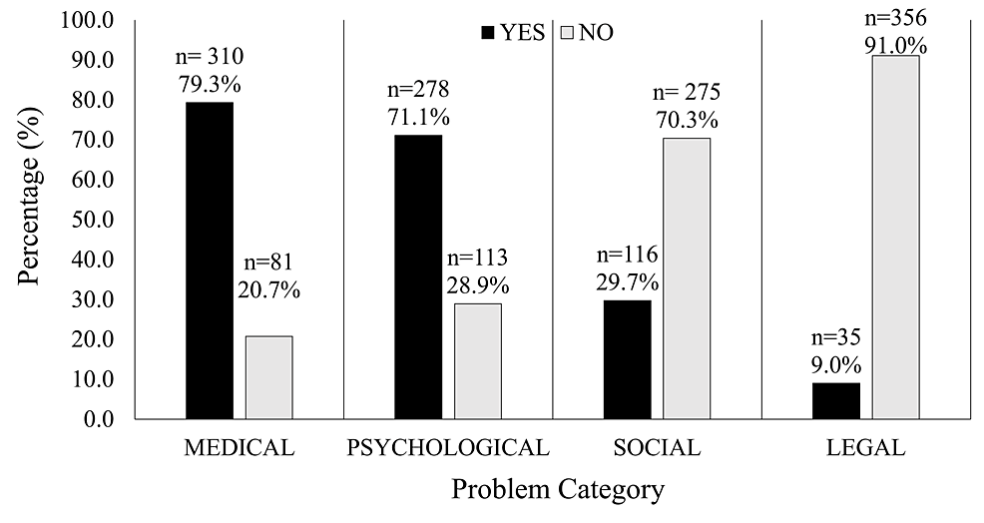
Problems reported by participants

When asked about problems faced during pregnancy, only 31 participants (7.9%) reported no problems of a biomedical, psychological, social, or legal nature, compared to 360 (92.1%) who reported experiencing some issues are further detailed in Figure 1. All types of problems were more prevalent in non-students.

Medical Problems

**Figure 2:**
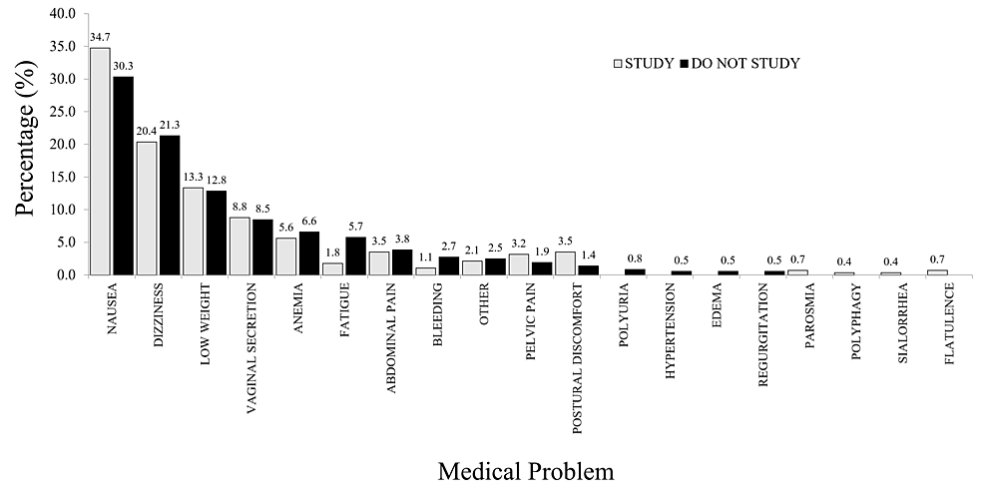
Medical problems reported by participants

Medical issues were associated with many symptoms developed during pregnancy or lactation (Table 5). Three hundred and ten adolescents reported at least one medical issue, with 651 symptoms. More symptoms were reported by non-students (n = 366, 56.2%) than students (n = 285, 43.8%). Conversely, of the 81 adolescents who reported no medical problems, 36 (44.4%) were non-students, and 45 (55.6%) were students. Figure 2 provides an overview of the main medical symptoms.

**Table 5:** Participant’s problems and studies

Type of Problem		Study		Do Not Study		Total		P-Value
		n	%	n	%	n	%	
Medical	Yes	142	36.3	168	43.0	310	79.3	0.118
	No	45	11.5	36	9.2	81	20.7	
	Total	187	47.8	204	52.2	391	100	
Psychological	Yes	127	32.5	151	38.6	278	71.1	0.183
	No	60	15.3	53	13.6	113	28.9	
	Total	187	47.8	204	52.2	391	100	
Sociofamiliar	Yes	57	14.6	59	15.1	116	29.7	0.736
	No	130	33.2	145	37.1	275	70.3	
	Total	187	47.8	204	52.2	391	100	
Legal	Yes	10	2.6	25	6.4	35	9.0	0.017
	No	177	45.3	179	45.8	356	91.0	
	Total	187	47.8	204	52.2	391	100	

Social Problems

**Figure 3:**
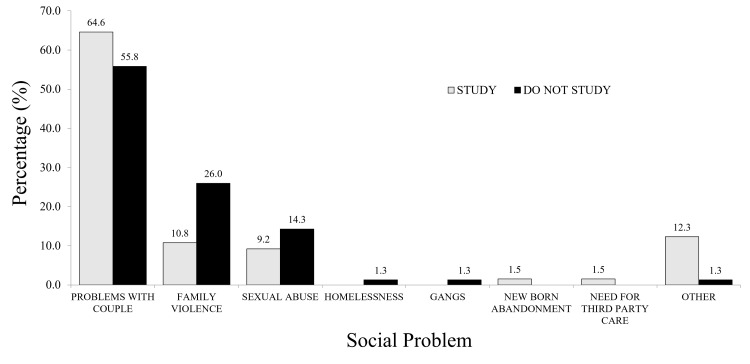
Social problems reported by participants

Most social problems were reported by non-students (52.2%), with a smaller proportion reported by students (45.8%). A similar pattern was observed among the 275 participants who reported no social problems, with 52.7% being non-students and 47.3% being students, although social problems were more prevalent among non-students. Social problems are detailed in Figure 3.

School Peers' Attitude Regarding the Pregnancy or Maternity of Their Classmate

Table 6 shows that most participants (55.2%) reported receiving unconditional support from their school peers to continue studying. This support was found among students (31.2%) and non-students (24.0%).

**Table 6:** School peers' attitude regarding the pregnancy or maternity of their classmate

Attitude Type	Peer Attitude	Study		Do Not Study		Total		p
		n	%	n	%	n	%	
Positive	Unconditional support	122	31.2	94	24.0	216	55.2	0.001
	Partial support	33	8.4	46	11.8	79	20.2	
	Subtotal	155	39.6	140	35.8	295	75.4	
Neutral	Didn´t know	24	6.1	44	11.3	68	17.4	
	Other	4	1.0	2	0.5	6	1.5	
	Subtotal	28	7.1	46	11.8	74	18.9	
Negative	Indifference	3	0.8	14	3.6	17	4.3	
	Bullying	1	0.3	3	0.8	4	1.0	
	Isolation	0	0.0	1	0.3	1	0.3	
	Subtotal	4	1.0	18	4.6	22	5.6	
	Total	187	47.8	204	52.2	391	100	

Family Attitudes During Pregnancy and Motherhood

As shown in Table 7, the family initially reacts with anger upon learning about their daughter's pregnancy. This initial negative reaction gradually shifts towards acceptance (transitional attitude 59.8%). Subsequently, a positive response from the family is evident in 35.3%, divided into acceptance (28.9%) and permanent support (6.4%). A negative family attitude is observed in a smaller group, constituting 4.9% (permanent anger 1.8%, indifference 1.5%, and other negative reactions 1.5%). There are statistically significant differences (p = 0.001) in the family's attitude towards pregnancy between girls who are studying and those who are not studying.

**Table 7:** Family attitudes during pregnancy and motherhood

Familiar Attitude		Study			Don't Study		Total		p
		n	%	n		%	n	%	
Transitional	Initial anger and then acceptance	128	32.7	106		27.1	234	59.8	0.001
	Subtotal	128	32.7	106		27.1	234	59.8	
Positive	Acceptance	34	8.7	79		20.2	113	28.9	
	Permanent support	14	3.6	11		2.8	25	6.4	
	Subtotal	48	12.3	90		23.0	138	35.3	
Negative	Permanent anger	3	0.8	4		1.0	7	1.8	
	Indifference	3	0.8	3		0.8	6	1.5	
	Other	4	1.0	2		0.5	6	1.5	
	Subtotal	10	2.6	9		2.3	19	4.9	
Total		186	47.6	205		52.4	391	100.0	

**Figure 4:**
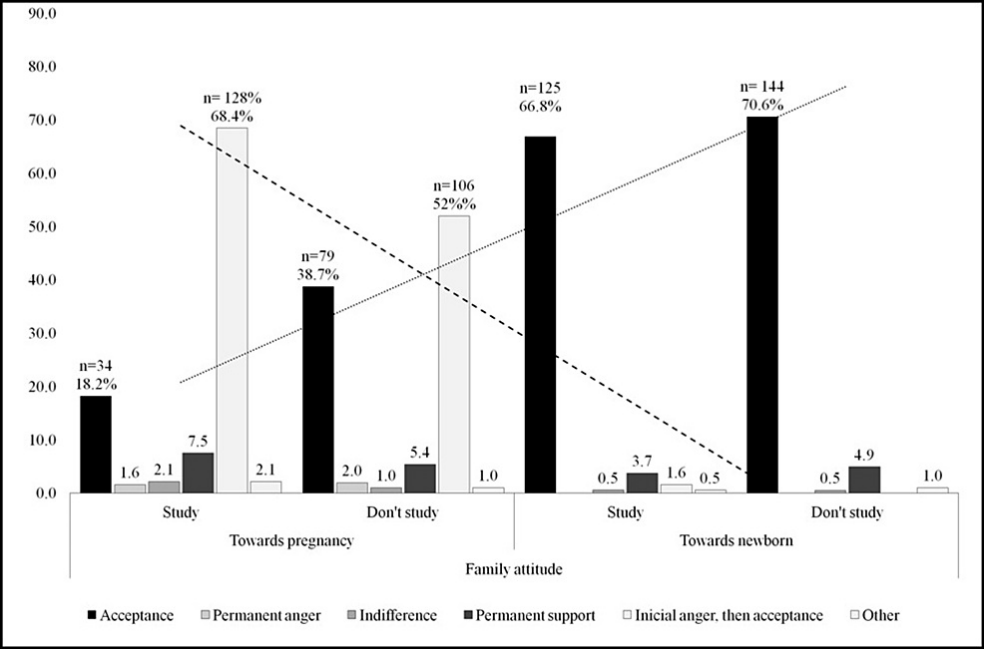
Family attitude tendency towards pregancy and newborn

Figure 4 indicates a trend of increased family support following the child’s birth compared to during the pregnancy. The degree of acceptance towards the daughter during pregnancy (8.7%) significantly increases (p < .001) once the grandson is born (32%), and the initial anger progressively decreases toward acceptance. This trend signifies a shift in family attitudes.

Legal Problems

**Figure 5:**
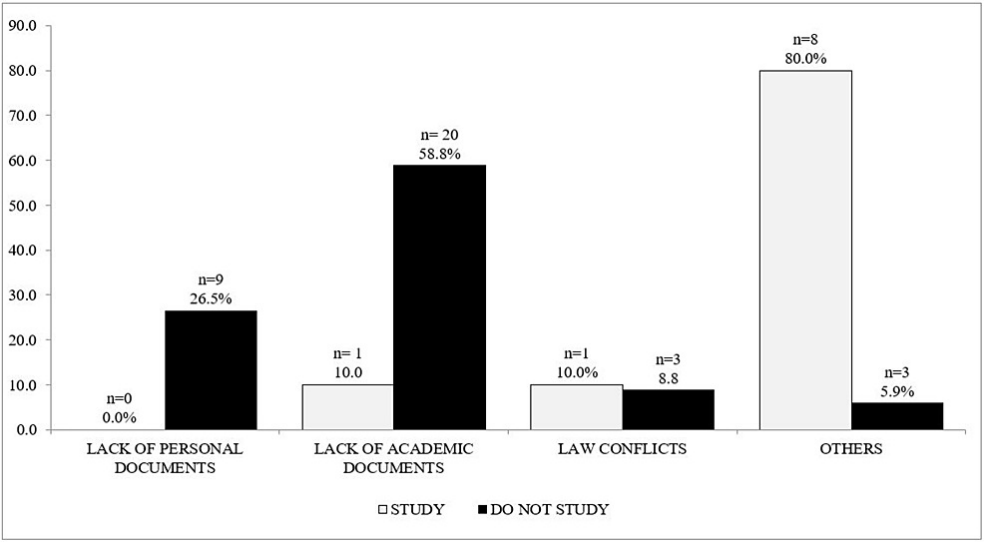
Type of legal problems

Legal problems ranked fourth in frequency (see Table 5). Among the 35 adolescents who reported legal problems, 44 issues were identified, averaging 1.25 problems per participant. Most of these (n = 35, 77.3%) occurred among non-students, with 10 issues (22.7%) reported by students (p= 0.017). Figure 5 highlights the main legal problems, illustrating that non-students faced more legal problems during pregnancy than students.

Qualitative analysis

In the qualitative analysis, focus groups were conducted where participants discussed their pregnancy experiences. The groups were segmented based on participants who had discontinued their education and had unwanted pregnancies despite not using contraceptives. The prevailing sentiment was one of the challenges: most participants had stopped their studies due to their pregnancies, felt isolated in their single status, struggled with accepting their pregnancies, experienced fear, resisted their new roles, and expressed a longing to resume their education.

Medical problems

Medical complications associated with pregnancy, such as nausea, dizziness, altered sleep and appetite patterns, and fatigue, often contribute to adolescents’ decision to discontinue schooling. These physiological changes can not only impact the well-being of the expectant adolescent but also disrupt the learning environment, potentially leading to intolerance among other students.

"S," a participant, shared her personal struggle with these issues: “… when I had my baby, I just kept vomiting, I don’t know why, but right now I have problems, they tell me my baby is very small, and I soon I will give birth, and I don’t want anything to happen to me, because I can have problems to deliver, and I don’t want to lose him either.”

"G" also echoed the impact of pregnancy symptoms on her school experience: “I left school in 5th level because I found out that I had two months of pregnancy, my belly barely showed, then I left. Before that, I was very sleepy, it made me want to sleep on the desk …”. She further detailed how the physical manifestation of her pregnancy led to intolerance from others: “… at the beginning I was starving and disgusted, and sometimes people who noticed it were bad because boys who were older than us, they bullied, they said many things, there were other students who were also pregnant, who dropped out of school because of them, they told us many things, but well, at first, they didn’t bother me, because we were closer. I was very sleepy, I slept well, but I was always sleepy.” Her account provides a firsthand perspective of the pressures pregnant adolescents face in educational settings.

Education-related problems

Educational institutions often struggle to adequately support pregnant students, who may face challenges not experienced by the general student body. For instance, physical discomfort can become a significant problem due to standardizing school furniture unsuitable for pregnant or lactating bodies. Educational institutions must adapt their environments, curricula, and methodologies to cater to the unique needs of pregnant students, ensuring they can pursue their education without interruption. This includes training teachers to understand frequent bathroom or medical breaks, incorporating comfortable furniture, and allowing time for physical activities like walking and stretching.

"A" recounted her personal encounter with this issue: “...yes, I had a hematoma, they told me that I couldn’t be in school because of that, so that’s also why I dropped out of school... at school, they helped me with the workshops, but later when everything was fine, when I no longer had the hematoma, they already told me to get out.”

Moreover, for many, the fear and stigma associated with a first pregnancy and a lack of information can lead to increased psychological and obstetric risk and create barriers to education. "M" shares: “When I found out that I was pregnant, I didn’t want to, I wanted to have an abortion... I just spent crying every night because I let me down, at school also, I no longer went out with my friends, seeing them go out and not me, so I was in that situation, it was also when everyone found out, it was kind of weird.” Crucially, schools must take action to prevent stigmatization and mistreatment of pregnant students and young mothers, ensuring their fair treatment and progress within the educational system.

Social problems

Pregnancy among young women can often lead to social challenges, including a lack of acceptance by family and friends. Pregnant students may experience heightened exposure to social scrutiny and feel a sense of shame among their peers and other educational community members, even while receiving conditional support from their families.

"D" provides her experience: “I have support from my mom and dad. All my relatives never told me to stop studying, they supported me, they told me that nothing was going to be like before... you can no longer go to meetings or outings with friends because you have a responsibility that is yours.”

"C" adds: “When I got pregnant, I felt I disappointed myself; in this case, I was afraid of my mom, and at first they found out, and everyone went against her, and then I felt good; I saw everyone’s support until this moment.”

An interesting factor emerged among a subset of migrant adolescents participating in the research. Their pregnancies were often a voluntary choice to affirm their femininity and life plans through motherhood and couple formation, as "E" shared: “I arrived from Venezuela a year ago; I came with my partner ... I sold candies on buses. He got a job, and then I got pregnant. My family reacted well because I lost a baby one year ago; my baby was two months old and passed away. My family was happy, excited, and I depend financially on my partner.”

## Discussion

Key findings

In general, many pregnant adolescents face pregnancy complications, with problems extending to psychological, social, legal, and biomedical domains. These adolescents predominantly hail from public schools and attend in morning and evening schedules in on-campus modalities, were in a civil union, and were primarily involved in housework. Unwanted pregnancies were common, though contraceptive methods were largely absent. A smaller subset had no occupation or were unemployed. An interesting observation was their stronger inclination towards pregnancy, signifying a life project directed towards motherhood rather than education. As it has been described poverty raises expectations for adolescent childbearing [17]. These individuals developed positive feelings about their pregnancies sooner than their counterparts who continued to study. Many had abandoned school before pregnancy, often due to migration. The absence of alternative life projects made motherhood highly valued among these adolescent mothers [18].

In contrast, families of adolescents who did not continue their education were often unaware or indifferent to the pregnancy, compared to those of studying adolescents. This suggests a dysfunctional family dynamic or an acceptance of a female life project centered on motherhood rather than education. Non-studying pregnant adolescents were more susceptible to domestic violence, sexual abuse, and risks associated with street life or gangs. Research reveals that sexual and physical abuse heightens the risk of early pregnancies over emotional abuse and neglect [19].

Non-studying pregnant adolescents were also more likely to suffer from mental health disorders, including anxiety, depression, and suicidal ideation, than those who continued their studies. The opportunity to continue education served as a critical protective factor for the mental well-being of these young mothers, in contrast to the variable results of education as a protective factor for mental health in other studies [20,21].

On the other hand, the profile of pregnant adolescents or mothers who maintained their studies typically included attendance at public schools, mostly in on-campus modalities. Some adopted blended and distance learning, with schooling predominantly in the morning, afternoon, and, importantly, at night. Most remained single, with very few forming civil unions with their partners, and their primary focus was on studying.

These young women tended to face more conflicts with their partners, greater challenges, and risks in baby care, thus necessitating help from third parties. Their desire for pregnancy was low as their life project was linked to education and professional development. Pregnant young women who continued their education had greater family support but also experienced negative reactions (e.g., fear, rejection) more frequently than their non-studying counterparts.

Interpretation

Adolescent pregnancy signifies a significant multifactorial phenomenon due to its high prevalence, multiple causes, and diverse negative impacts on young women, their partners, families, and society [22]. It disrupts the linear model of transitioning into adulthood-study, work, and success-imposed by societal norms. The Ecuadorian government, aiming to guarantee educational rights, mandates public education to be accessible and adequately funded.

However, our research reveals that numerous adolescents did not align their life projects with this socially desirable model. Instead, they affirmed their identity as women through motherhood or partnership formation, whether consciously or not. Gender stereotypes, an educational system that offers limited life project options, and an ambiguous societal message underpinning early pregnancy complicate these young women’s journey, generating several biopsychosocial consequences that stigmatize early pregnancy as unnatural or inappropriate.

Generalizability

This study gives a comprehensive approach, blending qualitative and quantitative methods to thoroughly investigate factors influencing school dropout among pregnant adolescents. However, the cross-sectional design limits the establishment of causal relationships and acknowledged challenges, including potential subjectivity in qualitative data and issues related to recall bias, suggest areas for improvement.

Our study had several important limitations. First, the participant enrollment was primarily conducted through qualitative meetings, which might have induced some selection bias. Second, our research was conducted before the COVID-19 pandemic, a fact that significantly limited data access and delayed the overall project execution. The effects of the pandemic on adolescent pregnancy and related experiences remain unexplored in our study. Additionally, we focused on public schools and on-campus modalities, potentially overlooking the experiences of adolescents from other educational contexts. Moreover, we did not account for the possible influence of cultural, socioeconomic, or regional variations on the experiences of pregnant adolescents. Lastly, the study relies on self-reported data, which might have introduced response bias, and the cross-sectional design precludes conclusions about causality.

The future direction of this study should focus on implementing and evaluating comprehensive support programs for pregnant adolescents and young mothers. These programs should go beyond traditional approaches and incorporate a multidisciplinary framework that considers societal, educational, and individual factors.

## Conclusions

Adolescent pregnancy, as observed by our study, proves to be a complex topic deeply entwined with societal norms, educational systems, and individual choices. The multifaceted nature of this phenomenon necessitates a more nuanced understanding and the development of strategies that respect the autonomy and identities of the young women involved. Our research, exploring many factors, that include marital status, nationality, occupation, and education characteristics, uncovered significant findings regarding pregnancy desire, contraception, reactions to pregnancy, and the prevalence of problems during this critical life stage. Addressing the urgent need to tackle systemic problems that hinder opportunities and expose young women to stigmatization and risk, the study advocates for a comprehensive approach. While acknowledging the challenges faced by adolescent mothers, underscores the importance of recognizing their resilience, growth, and the validity of their choices. Future efforts should prioritize holistic support, empowering these young women to navigate motherhood and educational and professional aspirations.

## Appendices

QUESTIONNAIRE Nº:

DROPOUT OF SCHOOL AMONG PREGNANT ADOLESCENTS AND MOTHERS WHO ATTEND THE COMPREHENSIVE CARE SERVICE FOR ADOLESCENTS (SAIA) OF QUITO ECUADOR, YEAR 2019

Objective: This is an anonymous, voluntary, and confidential questionnaire that aims to diagnose the biopsychosocial factors that promote school dropout of pregnant adolescents and mothers who attend the Service of Comprehensive Care for Adolescents (SAIA) at the "Isidro Ayora" Gynecological Obstetric Hospital in Quito.

The information obtained is very important to make recommendations for the benefit of the population of adolescents and youth of our country, for which we ask you to respond as seriously and honestly as possible.

Instructions: Mark with an X in the multiple choice questions:

**Table 8:** Survey applied to participants

1. GENERAL INFORMATION				
1.1 Archive number				
1.2. Date				
1.3 Age				
1.4 Marital Status	Single		Married	
	Civil union		Separated	
	Widowed		Divorced	
1.5 Relationship	Estable		Inestable	
	None			
1.6 Occupation	Domestic worker		Private employee	
	Public employee		Student	
	Own business		Family business	
	Housework		Unemployed	
	Other		None	
1.7 Economic dependence	Self-sufficiency		Partner	
	Parents		Parent-in-law	
	Others			
1.8 Nationality	Ecuadorian		Other	
1.9 Who do you live with?	Alone		Couple	
	Parents		Parent-in-law	
	Friends		Other family	
	Other (no family)			
1.10 Income	Less than 1 minimum salary (MS)		1-2 MS	
	3-4 MS		More than 4 MS	
2. PREGNANCY CHARACTERISTICS				
2.1 Gestational age		(weeks)		
2.2 Desirable pregnancy	Yes		No	
	I don't know			
2.3 Why did you want to get pregnant?	Motherhood desire		Marital desire	
	For company		To leave home	
	For curiosity		Fear of not being able to have a child	
	Partner's love		For imitaition someone	
	Recover a previous loss		In order not to lose my partner	
	Other			
2.4 If you didn't want to get pregnant. Why did you get pregnant?	I didn't take care		I didn't make the best decision	
	Contraception failed		I didn't know it could happen	
	I thought he took care		Someone abused me	
	Housework		He wanted to have a child	
	My family wanted me to have a child		Because my friends pressured me	
	Everybody does it		I was foolish	
	Other			
2.5 Did you use any contraceptive method?	Yes		No	
2.6 If you used a contraceptive method. Which was?	Douching		Condoms	
	Emergency contraception		Pills	
	Monthly injections		IUD	
	Coitus interruptus		Rhythm method	
	Implant		Natural infusions	
	Hormone patches		Spermicides	
	Vaginal ring		Cervical mucus	
	Other			
2.7 Reaction to pregancy	Acceptance		Guilty	
	Fear		Hope	
	Anger		Rejection	
	Indifference		Other	
2.8 Did you think about abortion?	Yes		No	
2.9 Did you try to abort?	Yes		No	
2.10 Family reaction to pregnancy	Acceptance		Permanent anger	
	Indifference		Abuse	
	Permanent support		Initial anger and then acceptance	
	Abandonment		Other	
2.11 Type of family support	Economic		Prenatal care	
	Educational support		Employment support	
	Legal		Other	
3. MOTHERHOOD PREGNANCY				
3.1 Gestational age				
3.2 Child's age				
3.3. Number of children				
3.4 Type of delivery	Vaginal		Assisted vaginal	
	C-section		Other	
3.5 Newborn condition at birth	Healthy		Sick	
3.6 Newborn current condition	Healthy		Sick	
	Gets sick all the time			
3.7 Family reaction to newborn	Acceptance		Reject	
	Indifference		Permanent support	
	Initial anger and then acceptance		Abandonment	
	Other			
3.8 Self-satisfaction for motherhood	High		Medium	
	Low		None	
3.9 Family support with motherhood	None		Emotional	
	Economic		Child care	
	Educational support		Employment support	
	Legal support		Other	
3.10 Who takes care of the child daily?	Myself		Partner	
	Parents		Parents in law	
	Other relatives		Babysitter	
	Kindergarten		Hospital	
	Other			
4. EDUCATIONAL CHARACTERISTICS				
4.1 Last course approved				
4.2 Level	Elementary		High School	
	Technical career		Craft career	
	Other			
4.3 Type of school	Public		Private	
	Municipal		Semi-public	
	Foundation		Other	
4.4 School modality	On-campus		Blended	
	Distance		On line	
	Other			
4.5 Time of study	Morning		Afternoon	
	Night		No schedule	
	Other			
4.6 Are you currently studying?	Yes		No	
4.7 How long ago did you stop studying?				
4.8 Why did you stop studying?	I don't like studying		Poor school performance	
	Behavior problems		Pregnancy	
	Economic problems		Marriage or union	
	Problems with partner		Legal problems	
	Child care		Family problems	
	Illness		I finished the career	
	Migration		Boredom	
	Peer bullying		Teacher bullying	
	Shame		Gangsters	
	Other			
4.9 Who decided to stop studying?	Myself		Partner	
	Father		Mother	
	Other relatives		Parents in law	
	Parent's couple		Student Counseling Department	
	School's director		Teacher	
	School's inspector		Doctor	
	Other			
4.10 Partner attitude about your studies	None support		Unconditional support	
	Anger		Partial support	
	Indifference		Bullying	
	Jealousy		Devaluation	
	Other			
5. PREGNANCY AND SCHOOL DROPOUT				
5.1 Medical problems during pregnancy	Nausea		Dizziness	
	Low weight		Anemia	
	Parosmia		Polyphagy	
	Sialorrhea		Polyuria	
	Postural discomfort		Fatigue	
	Vaginal secretion		Bleeding	
	Hypertension		Edema	
	Abdominal pain		Hydrorrhea	
	Flatulence		Regurgitation	
	Pelvic pain		Other	
	None			
5.2 Psychological problems during pregnancy	Anxiety		Depression	
	Irritability		Aggressiveness	
	Lack of self-control		Suicidal ideation	
	Adaptative disorder		Shame	
	Alcohol abuse		Substances abuse	
	PTSD		Suicide attempt	
5.3 Social problems	Couple's problems		Family violence	
	Sexual abuse		Promiscuity	
	Sexual exploitation		Homelessness	
	Gangs		Newborn abandonment	
	Need for third-party care		Other	
	None			
5.4 Legal problems	Lack of legal documents		Lack of academic documents	
	Non valid academic documents		Law conflicts	
	Other		None	
5.5 Pears attitude towards pregnancy	They didn't know		Unconditional support	
	Anger		Partial support	
	Indifference		Bullying	
	Isolation		Harrassment	
	Other			
6. SCHOOL ATTITUDE TOWARDS PREGNANCY				
6.1 Did your school find out you were pregnant?	Yes		No	
	I don't know			
6.2 School attitude towards pregnancy	Rejection		Support	
	None		I don't know	
6.3 Did the school take any of these actions in response to your pregnancy?	Temporary suspension		Expulsion	
	Grades sanction		Behavior sanction	
	Change in school schedules		Homework flexibility	
	Schedule flexibility		Evaluations flexibility	
	Not wearing student uniform		Psychological support	
	Ease of transfer to another school		Exemption of subjects	
	Normal studies		Exclusion in special programs	
	Overprotection		Other	
7. DESIRE FOR EDUCATIONAL REINSERTION				
7.1 Do you want to go back to study?	Yes		No	
	I don't know			
7.2 In how long?		months		
7.3 What would you like to study?	Technical high school		Regular high school	
	University		Courses for work	
	Others			
7.4 What would be the conditions to return to study?	Help in childcare		Parent's help	
	Homework flexibility		Schedule flexibility	
	Evaluations flexibility		Psychological support	
	Change in school modality		Exemption of subjects	
	Short duration of studies		Vocational orientation	
	Economic support		I don't know	
